# Supplementary material for: Disparities in Survival Outcomes among Racial/Ethnic Minorities with Head and Neck Squamous Cell Cancer in the United States
Source: Cancers (Basel). 2023 Mar 15;15(6):1781. doi: 10.3390/cancers15061781 (PMC10046381; doi:10.3390/cancers15061781)

Supplemental Table S1: Propensity Score Matching Analysis with Pair-wise Comparisons of Race

	N	HR	p-value
Asian vs White (Ref)	10,506	0.87 (0.82 0.92)	<0.0001
Asian vs Black (Ref)	10,506	0.73 (0.69 0.77)	<0.0001
Black vs White (Ref)	29,724	1.13 (1.09 1.16)	<0.0001

Supplemental Figure S1. Overall Survival for Oropharyngeal HNSCC patients by Race and Human Papilloma-Virus (HPV status). (A) HPV positive; (B) HPV negative

(A) HPV-Positive

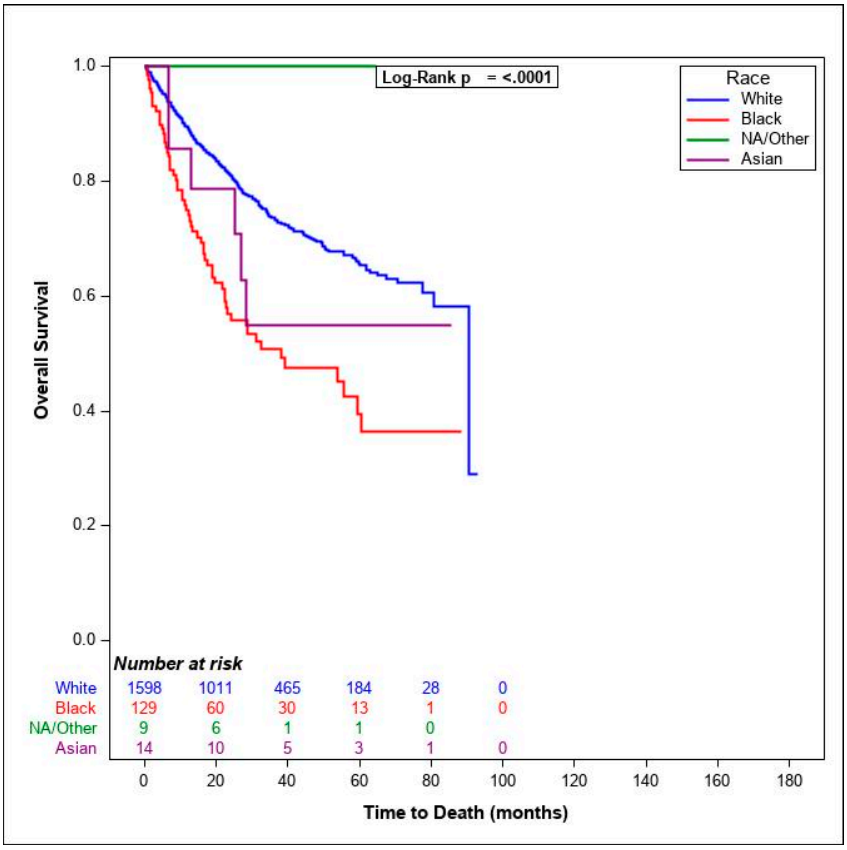

(B) HPV-Negative

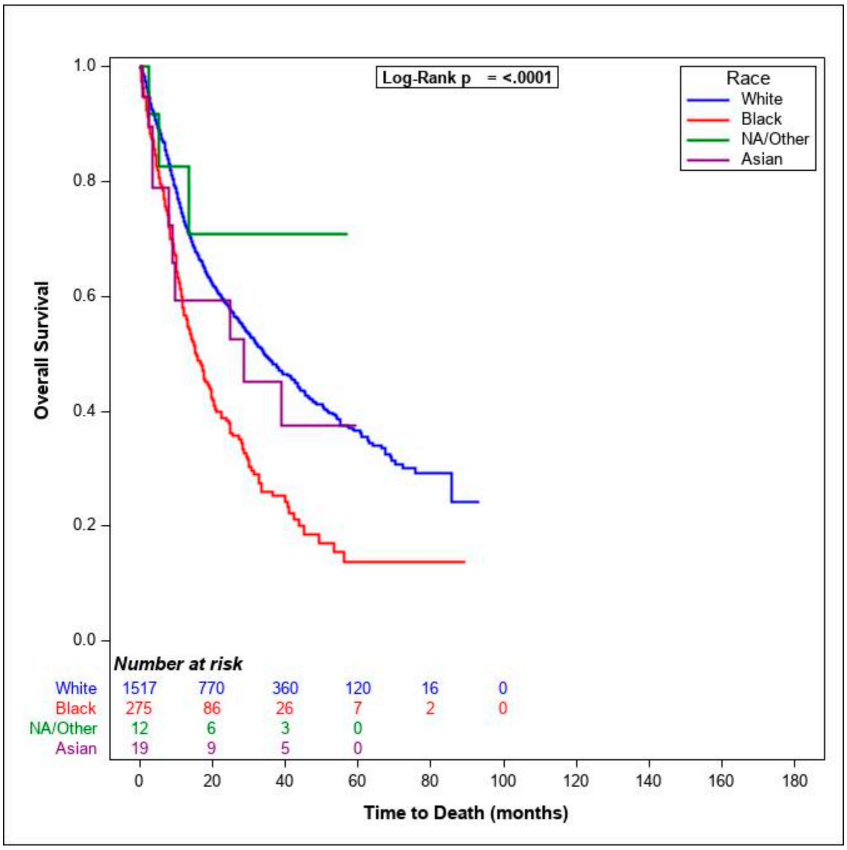

Supplemental Figure S2. Overall Survival for Black Head and Neck Cancer patients by (A) Income; (B) Insurance Status; (C) Receipt of Surgery

(A) Income

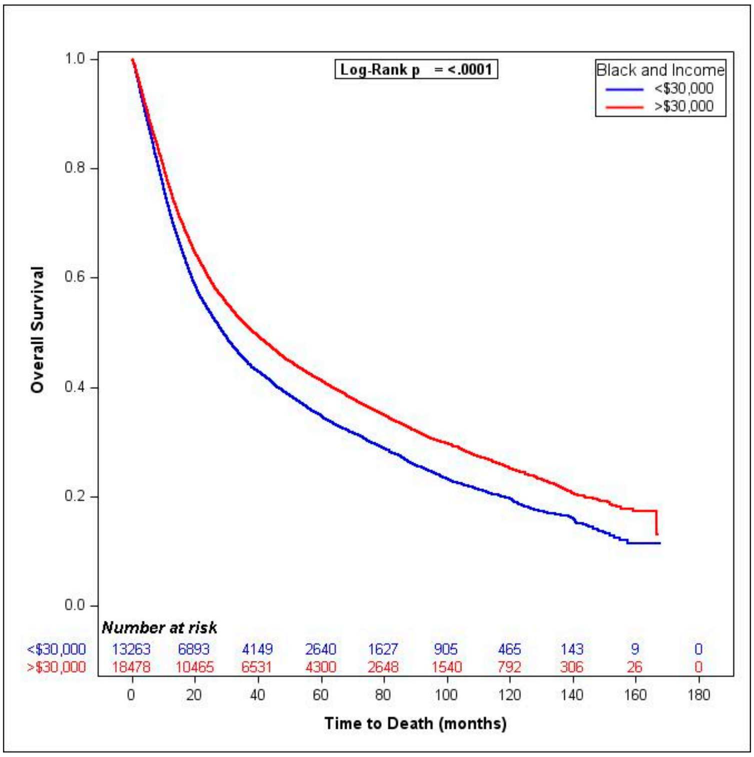

(B) Insurance Status

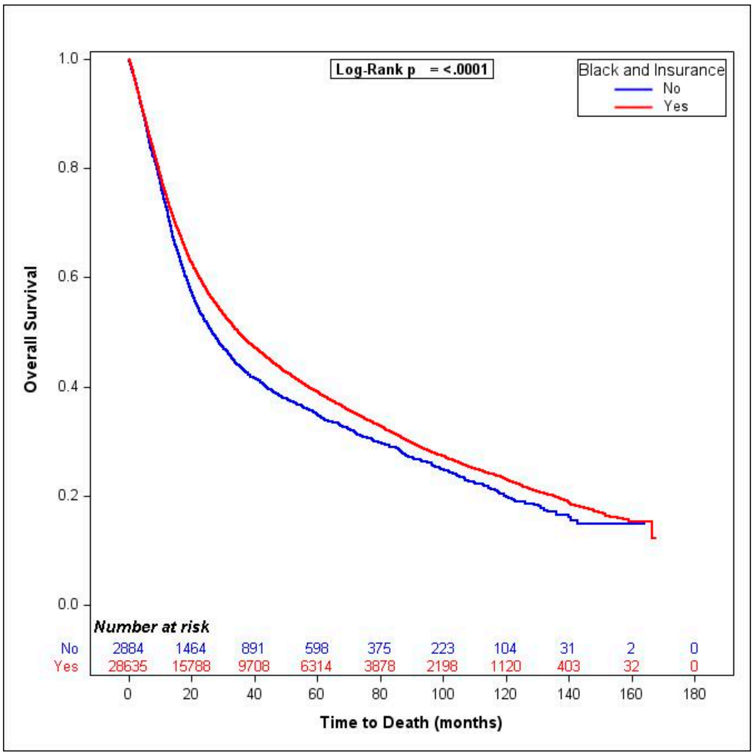

(C) Surgical Status

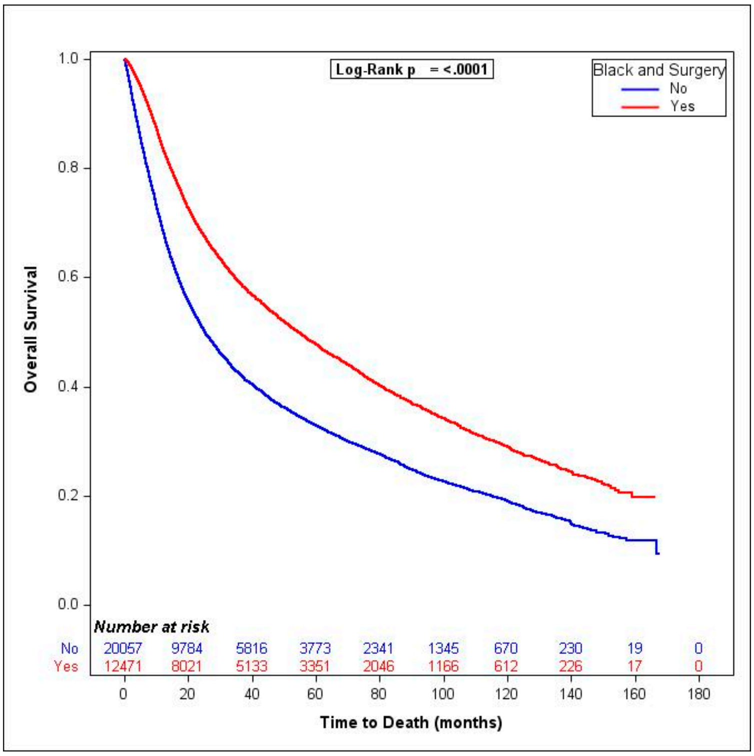

Supplement: Supplementary file 1 [file cancers-15-01781-s001.zip › cancers-2223632-supplementary.pdf]
